# Penile Gangrene and Necrosis Leading to Death Secondary to Strangulation by Condom Catheter

**DOI:** 10.1155/2018/3702412

**Published:** 2018-06-27

**Authors:** Youness Jabbour, Bilgo Abdoulazizi, Tarik Karmouni, Khalid El Khader, Abdellatif Koutani, Ahmed Iben Attya Andaloussi

**Affiliations:** ^1^Urology B Department, Ibn Sina Teaching Hospital, Rabat, Morocco; ^2^Faculty of Medicine and Pharmacy of Rabat, Morocco

## Abstract

Condom catheters are widely used in the management of male urinary incontinence, bedridden patients, and geriatric population. They are considered to be safe; however they are associated with serious complications in case of an incorrect use. We report a dramatic case of penile strangulation by condom catheter tardily discovered till occurrence of necrosis and gangrene leading to death in an elderly bedridden and diabetic man. Through this case we emphasize the importance of patient education for the correct use of condom catheters and remind care providers to maintain a high level of sensibility to complication generated from long-term use of condom catheters.

## 1. Introduction

Condom catheter (CC) is a noninvasive device used as an external drainage system in the management of urinary incontinence in men but also in patients with reduced mobility or bedridden.

These catheters are known to be discrete, reliable, comfortable, and very easy to use which makes them preferable to bladder catheter, especially since certain types of CC can also reduce the risk of urinary tract infections compared with indwelling catheters [1, 2].

Even if CC are less invasive, their use is not completely denuded of risks and particularly if mishandled.

Several cases have been reported in literature, some of them describing serious injuries such as necrosis, gangrene, and amputation of the penis [3–6].

We report a fatal evolution of penis strangulation by condom catheter.

## 2. Case Presentation

A 72-year-old patient with a past medical history of poorly managed diabetes on oral antidiabetic agents and a recent history of ischemic stroke with left sequential hemiparesis forcing him to be bedridden. He lives with his family, helped by his daughter. He had a condom catheter for three months. He presented to the emergency department complaining of a swollen and blackish genital area, being febrile, and deterioration of his general condition.

The general examination found an altered patient, febrile, with tachycardia and low blood pressure at 80/50 mmHg.

**Figure 1:**
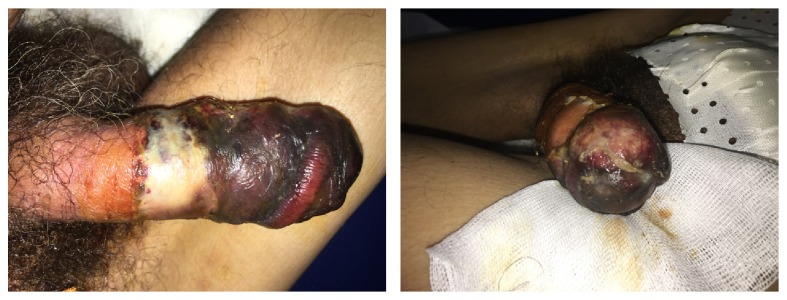
Penile aspect at the admission after removing the condom catheter.

Clinical examination revealed a smelly genital area with purulent urine drops from urinary meatus and a gangrenous lesion of the zone covered by the condom catheter and predominance of necrotic and ulcerous plaque of the distal penile suggesting penile strangulation with condom catheter (Figure 1).

Our patient confirmed that he reinforced the adhesive tape of the condom catheter to fix an urinary leakage that occurred during the last change of his condom catheter indwelling for eight days.

Biology found a high rate of white blood cells and a high CRP. A cystostomy was performed, bringing back 400 ml of purulent urine whose bacteriological study isolated* E. coli*.

The patient underwent a debridement under wide-spectrum antibiotics (aminoglycoside, metronidazole, and ceftriaxone). Unfortunately, he did not recover from his septic shock and died within four days of admission.

## 3. Discussion

Penile strangulation is a rare urologic emergency with potentially severe clinical consequences reported to occur most often during manoeuvres aiming to obtain sexual pleasure or prolonged erection in adults [7].

Its occurrence due to condom catheter is even more rare, reported only few times in literature [3–6].

The clinical presentation depends on the degree and duration of strangulation.

Thus, firstly, edema and venous stasis of the distal portion of the penis occurs associated with a decrease in cutaneous sensitivity. Then ulceration followed by cutaneous necrosis at below strangulation level. The longer the penile strangulation persists, the more the arterial flow is also compromised, resulting in ischemia and gangrene of the penis.

Strangulation, inflammation, or other discomfort may not be detected or reported by the patient as was the case for our patient.

Lack of nociceptive feedback in diabetic patients makes patients unaware of painful sensation due to the tourniquet effect of the CC. Consequently, early symptoms of possible complications due to CC can be overlooked.

Complications and incidents of CC use remain underestimated. Users of CC should be aware of those complications and informed about precautions to take to avoid them.

Given the fatal ending of our patient we find it worthy to review some important precautions to take when using condom catheters.

To ensure a comfortable and secure fit, it is very important to get the appropriate size of the condom catheter.

It is necessary to measure the penile circumference. The measurement should be taken from the penile base where the diameter is the largest to estimate the correct size. It can sometimes be difficult to choose the right size of condom catheter if the measurement is between two sizes. In this case, it is advisable to choose the smallest size. The material of the condom catheter is flexible enough to allow the condom catheter to be well adjusted, without being too tight. Choosing the largest size could result in urinary leakage [8].

Before applying the condom catheter, the penis should be cleaned with a neutral pH value soap and water and then dried to allow a tight fixing of the adhesive and an eviction of leaks.

Pubic hair should be trimmed away from the base to the penis to stop it from sticking at the condom catheter.

It is also important to note that the adhesive tape of the condom catheter at penile base is not applied too tightly. The skin of the penis should be inspected routinely after applying the condom catheter to assure that the catheter is not placed too tightly [3, 5].

The applied condom catheter should be changed every 48 hours. When replacing the condom catheter, it is important to carefully examine the skin covered by the device.

Most often CC users face problems of dislodgement and leaking due to unsuitable size or poor positioning. This problem seems to be solved with the use of adhesive strips to avoid urinary leakage. There is also the occurrence of cutaneous lesions and allergies and urinary tract infection due to long-term use of CC.

Ouslander et al. noticed a significantly higher incidence of urinary tract infection in men with condom catheter worn continuously than among continent or incontinent patients without condom catheter [9].

Different materials are used in confection of condom catheters. It seems that silicone probably has an advantage of its cutaneous tolerance making rare allergic reactions as well as its translucent nature offering a good visibility of the skin to identify any irritation or appearance of skin problem [8].

Our case represents a fatal evolution of penis strangulation by condom catheter that, to our knowledge, had never been reported before in literature. It also highlights the importance of careful nursing care and assistance when condom catheter is placed for urinary drainage to achieve the best results and prevent complications.

Patients and care providers should be aware about the correct technique of wearing a condom catheter and also have an increased awareness to detect early signs of infection or gangrene.

Early presentation in the course of a strangulation increases the chances of saving the penis just by removing the condom catheter.

Although being a rare complication of condom catheter use, strangulation can have serious consequences and should always be kept in mind when using condom catheters.
